# Supplementary material for: Chromothripsis during telomere crisis is independent of NHEJ, and consistent with a replicative origin
Source: Genome Res. 2019 May;29(5):737–49. doi: 10.1101/gr.240705.118 (PMC6499312; doi:10.1101/gr.240705.118)
Supplement: Supplemental Material [file supp_gr.240705.118_Supplemental_file_1.zip › contigs/annotated_contigs/DB53/contig.3.DB53_length_322_mean_cov_15.5621118012.docx]

**DB53_length_322_mean_cov_15.5621118012**

```
GACAGAGAAAGACAGAGACAAACACAGAGAGAAAGAAACAGAAAGAGAGAGAGAGAGAAAGAGACAGGAAGGGAGAGAGAGAGACAGAC
 >chrUn_gl000220:132344-132533 - E=6e-35 p=0e+00
AGACAGAGAAAGAGAATAAGACAGAAGACAGATGCAGTGAGACAGGCAGAGAGAGAGACAGAGAAAGACAGAGACAGACAGAAAGAGAG

AGACAGAGAGAA|AGAGA|GAAACAGAAAGGGAGGGAGAGAGAGACAGACAGACGGACAGGCAGAGAAGGAGAGTAAGACAGAAGACAG
             >chr2:133028733-133028810 + E=2e-20 p=2e-02
ACA|CAG|AAGAAAAACAACCACCAAGAAAGTTAAAATTCTCCAATGGTCGGAAGTTCA       >chr2:133033823-133033874 + E=3e-16
```
